# Covalently Modified Molecular-Recognition-Capable UV-Transparent Microplate for Ultra-High-Throughput Screening of Dissolved Zn^2+^ and Pb^2+^

**DOI:** 10.3390/s24144529

**Published:** 2024-07-12

**Authors:** Bálint Árpád Ádám, Bálint Kis-Tót, Bálint Jávor, Szabolcs László, Panna Vezse, Péter Huszthy, Tünde Tóth, Ádám Golcs

**Affiliations:** 1 Department of Organic Chemistry and Technology, Budapest University of Technology and Economics, Szent Gellért tér. 4, H-1111 Budapest, Hungary; adamb@edu.bme.hu (B.Á.Á.); javorb@edu.bme.hu (B.J.); panna.vezse@edu.bme.hu (P.V.); huszthy.peter@vbk.bme.hu (P.H.); 2 Department of Pharmaceutical Chemistry, Semmelweis University, Hőgyes Endre utca 9, H-1092 Budapest, Hungary; balint.kis-tot@edu.bme.hu; 3 Department of Inorganic and Analytical Chemistry, Faculty of Chemical Technology and Biotechnology, Budapest University of Technology and Economics, Műegyetem rkp. 3, H-1111 Budapest, Hungary; laszlo.szabolcs@vbk.bme.hu; 4 HUN-REN–BUTE Computation-Driven Chemistry Research Group, Műegyetem rkp. 3, H-1111 Budapest, Hungary; 5 HUN-REN Centre for Energy Research, Konkoly-Thege Miklós utca 29-33, H-1121 Budapest, Hungary; 6 Center for Pharmacology and Drug Research & Development, Department of Pharmaceutical Chemistry, Semmelweis University, Hőgyes Endre utca 9, H-1092 Budapest, Hungary

**Keywords:** UV absorbance, chemosensing, microtiter plate, zinc, lead

## Abstract

Zn^2+^ has a crucial role both in biology and the environment, while Pb^2+^ presents serious hazards in the same areas due to its toxicity, and the need for their analysis often exceeds available instrumental capacity. We report, herein, a new high-throughput optochemical screening method for Zn^2+^ and Pb^2+^ in various solutions. Moreover, we also introduced a new and generalizable three-step-microplate-modification technique, including plasma treating, linker-docking and photocatalytic copolymerization. The surface of a commercially available 96-well-cycloolefin-microplate was treated with atmospheric plasma, and then, the bottoms of the wells were covered by covalently attaching a methacrylate-containing linker-monolayer. Finally, the preactivated microplate wells were covalently functionalized by immobilizing bis(acridino)-crown ether-type sensor molecules, via photocatalytic copolymerization, to a polymethacrylate backbone. This sensing tool can be used in all microplate readers, is compatible with liquid handling platforms and provides an unprecedently fast monitoring (>1000 samples/hour, extrapolated from the time required for 96 measurements) of dissolved Zn^2+^ and Pb^2+^ among recent alternatives above the detection limits of 8.0 × 10^−9^ and 3.0 × 10^−8^ mol/L, respectively, while requiring a sample volume of only 20 µL.

## 1. Introduction

In recent years, chemosensors have gained indisputable significance in the detection of biologically and environmentally important cations, anions and neutral molecules due to their simplicity and low detection limits [1,2]. Their importance is illustrated well by the numerous review articles published in the last decade [1,2,3,4,5,6,7,8], which investigate the reported results from different perspectives, such as the diversity of fluorophores, receptor units, sensing mechanisms and analytes. Despite the considerable development in this field, challenges still exist, and new ones are always emerging with rapidly changing situations, providing the driving force for the continuous improvement of chemosensors. Such a driving force could be the reduction of the highest permitted limit of a metal ion in drinking water, for example, which requires the development of sensors with higher selectivity and sensitivity. In addition, future sensors must be cost-efficient, rapid and easy-to-use, which avoid the need for complex instrumentation. Moreover, regarding the sensor design, the suitability to modern technology, such as high-throughput applications, integrability with widely available instrumentation or portability should also be considered.

Among all the toxic heavy metals, lead is one of the most important as it is highly poisonous to almost all human organs [9], making it crucial to accurately detect its presence in the environment. In contrast, zinc, which has a relatively low toxicity, plays an essential biological role [10]; therefore, its selective measurement in the environment is pivotal as well. Concentrations of heavy metal ions in complex samples can generally be measured using various analytical methods, such as atomic absorption spectroscopy (AAS) [11], inductively coupled plasma techniques (ICP-MS [12], ICP-OES [13]) or anodic stripping voltammetry (ASV) [14]; however, these require a high level of instrumentation, and the sample preparation can be time-consuming.

In comparison, optical chemical sensors (optodes) are fast and easy-to-use alternatives. Traditional bulk optode membranes are plasticized polymers containing an ion-selective ionophore, proton-selective chromoionophore and lipophilic anionic sites [15,16,17,18,19,20]. When the membrane comes into contact with the sample containing the favored metal ions, an influx of metal ions takes place accompanied by the deprotonation of the chromoionophore, which results in optical signal changes. However, these conventional optosensor membranes have major drawbacks. One of the problems with bulk optodes is that their response time is usually too long, since a uniform distribution of components, limited by diffusion, is required to obtain the theoretically expected constant optical response [21]. Not only can this fact slow down the measurement, but it can also make the membrane regeneration and subsequent reuse complicated. To keep diffusion resistance low, thin membranes with a thickness of only a few micrometers with the addition of a large amount of plasticizer to the polymer are commonly used. However, this solution poses a further challenge, as it can cause the leakage of the photoactive component, which reduces the durability of the optode [21]. The loss of the ionophore can be most effectively avoided by its covalent immobilization to the polymer matrix. As an example, Liu et al. substituted the chloro groups of PVC with azide, and a crown ether-based ionophore was linked to this functionalized PVC via a click reaction [22]. These sensors showed improved selectivity and lifetimes, but also prolonged the equilibration and thus the response time. Another disadvantage of traditional optodes is that the ion exchange equilibrium responsible for the sensor signal is pH-dependent [23]. This means that parallel pH-measuring, or pH-buffering is often required. To overcome this limitation, the proton-selective chromoionophore can be omitted if the metal-ion-selective ionophore, which does not contain proton ionizable groups, has fluorogenic or chromogenic properties. As an example, Citterio et al. synthesized a crown ether-based fluorogenic Li^+^ ionophore and the corresponding optode was pH-independent in the range from 6 to 8 [24].

Measurements using optodes can not only be accelerated by reducing the response time of the sensors, but also by making them suitable for high-throughput screening (HTS). Typically, HTS techniques are microtiter plate-format assays as the plates with standard dimensions are compatible with automated liquid handling equipment. Kim et al. were the first to publish microtiter plate-format optodes, which were prepared by dissolving the components of the membrane in tetrahydrofuran (THF) and by uniformly dispensing this solution into each well of the microplate [25]. This membrane was selective for Na^+^, K^+^ and heparin. Using this method, they were able to achieve a throughput of ~100 samples in less than 5 min. Despite this useful feature, microtiter plate-format optodes are not widely used, for they still require the use of traditional membrane compositions and optodes. Further studies are reported for microplate-based membranes, which are selective for polyanionic species [26,27], polycations [28,29], F^−^ [30] and Cu^2+^ [31]. In addition, Hamilton et al. published a polymeric hydrogel-containing photoinduced-electron-transfer (PET)-based sensor for Zn^2+^ in 96-well microplate-format [32]. The concentration-dependent fluorescence intensity showed linearity in the range of $2.5\cdot 10^{-4}\;\mathrm{to}\;1.75\cdot 10^{-3}$ M, and the limit of detection (LOD) was $1.74\cdot 10^{-4}$ M. In one of the latest results of our research group, we recently reported a novel concept-based nonequilibrium-type Pb^2+^-selective optode for high-throughput monitoring with an LOD of $4\cdot 10^{-7}$ M [33].

In order to covalently attach molecules to microtiter plates, the polymer surface first has to be preactivated, as most of the commercially available polymers are not suitable for binding molecules. The polymer surface can be functionalized using various techniques, such as wet chemical treatment, organosilanization, plasmas, flames and UV-irradiation [34]. Unlike chemical methods, plasma treatment is a rapid, solvent-free and environmentally friendly technique [35]. Another advantage is that this treatment affects only the topmost layer of the polymer surface, leaving the properties of the bulk phase unchanged [35,36]. Furthermore, the chemical effect of the plasma is highly variable depending on the used gas [36]. Atmospheric pressure plasmas have further advantages compared to low-pressure ones. The treatment time is even shorter (typically a few seconds), the method does not require costly vacuum operations and therefore, it is easily adaptable industrially [37].

Microtiter plates made from cyclic olefin copolymer (COC) are desirable tools for spectroscopic measurements due to the high optical clarity and UV-transparency of the copolymer. Several studies report a low-pressure plasma treatment of COC using oxygen, argon or both [38,39,40]. The water contact angle (WCA) can be decreased from the initial ~90° to about 20° depending on the treatment time, the gas type and the applied plasma-intensity. This is felt to be the result of surface functionalization with *O*-containing functional groups, such as carbonyl, carboxyl, hydroxyl, etc. Along these lines, Bourg et al. applied atmospheric air pressure plasma on COC plates [41]. The WCA was decreased from 95° to 48° in 300 ms. Moreover, the treatment did not influence the transparency of the plate.

In this study, we wanted to develop a novel microtiter-plate-format high-throughput optochemical screening method for Zn^2+^ and Pb^2+^. Due to the microtiter plate basis, the device is compatible with laboratory automation, such as any type of liquid-handling equipment, plate transferring robots and plate readers, and it does not need advanced analytical instrumentation. An atmospheric-pressure-air-plasma-based procedure was developed to covalently attach a copolymer sensor membrane to the microtiter plate. The aim was to provide the regenerability and long-term durability of the device and the ability to measure organic solvents, as well as unusual chemicals or apolar media. Moreover, the reported general method, which enables the covalent functionalization of UV-transparent COC-microplates can be used for the future development of high-throughput separation and monitoring tools.

## 2. Materials and Methods

### 2.1. Materials and Instruments

Chemicals were purchased from Sigma-Aldrich Corporation (Saint Louis, MO, USA, owned by Merck, Darmstadt, Germany) and used without further purification, unless otherwise noted. A Greiner Bio-One UV-STAR^®^ 96-well (half area), clear, UV-transmittance (up to 230 nm) cycloolefin flat-bottom microtiter-plate (Greiner Bio-One Hungary Ltd., Mosonmagyaróvár, Hungary) was used for further functionalization.

**Figure 1:**
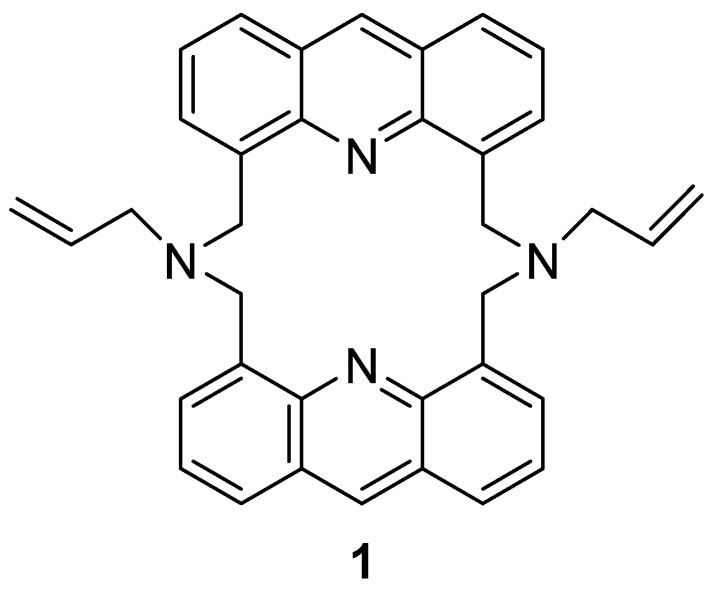
The applied Zn^2+^ and Pb^2+^-selective optosensor molecule (**1**).

A bisacridino-diaza-20-crown-6 ether-type macrocycle containing reactive allyl groups (**1**, see Figure 1) was used as an optical sensor molecule [42]. Covalent attachment to the preactivated microplate surface was performed via copolymerization with the allyl units.

This ionophore can fulfill receptor and signaling functions simultaneously. Selectivity was previously proved in solution phase [42]. The sensor molecule preferred Zn^2+^ (log*K* = 5.7) over the other 22 potentially competing cations (Rb^+^, Li^+^, Cs^+^, Mn^2+^, Fe^2+^, Ba^2+^, Sr^2+^, Hg^2+^, Cd^2+^, K^+^, Ni^2+^, Co^2+^, Na^+^, Cu^2+^, Ag^+^, Ca^2+^, Mg^2+^, Cr^3+^, Pd^2+^, Pb^2+^, Al^3+^, Bi^3+^) and showed a reversible complex formation with a 1:1 stoichiometry. The interfering ions were Al^3+^, Cd^2+^ and Pb^2+^. It was shown that the coordination of anions (H_2_PO_4_^−^, NO_3_^−^, HSO_4_^−^, CH_3_COO^−^, F^−^, Cl^−^, Br^−^, I^−^) did not take place even when the ionophore was protonated [42].

UV/Vis spectra were recorded on a TECAN Infinite^®^ 200 PRO UV-Vis microplate reader (Tecan Group Ltd., Männedorf, Switzerland) in absorbance scan mode (230–310 nm, 1 nm-step, 5 s integration time with 5 No of flashes). The sample volume was always 20 µL. Zn^2+^-acetate and Pb^2+^-nitrate were used for preparing all artificial samples. Spectroscopic measurements were carried out at room temperature (25 ± 1 °C). During spectrophotometric measurements, the solutions were pipetted into the wells containing the optosensing membrane. The spectra were corrected with the background noise and the dilution effect. The reported data points were the average values of at least three independent measurements. For evaluation and visualization of the spectroscopic results, OriginPro 8.6 (OriginLab Corp., Northampton, MA, USA) software was used.

pH measurements were carried out using a Mettler Toledo SevenEasy pH meter (Mettler Toledo, Columbus, OH, USA) fitted with a Mettler Toledo Inlab microelectrode. The different pH values were set using aqueous solutions of nitric acid or sodium hydroxide. The accuracy of pH measurements was found to be within ±0.1 unit.

The multielement composition of real samples was measured via inductively coupled plasma optical emission spectroscopy (ICP-OES). To the samples of 5 mL, aqueous nitric acid solutions (63 wt%) of 50 µL were added. The measurements were carried out in simultaneous, multielement mode using a Labtest Plasmalab ICP-spectrometer (Labtest Equipment Company, Valparaiso, IN, USA) equipped with a 40-channel Paschen-Runge vacuum polychromator with photomultiplier detectors. Further details regarding the instrumentation and measurement configurations are available in the Supplementary Materials.

In the case of the artificially contaminated samples, the Zn^2+^ and Pb^2+^ content were determined via atomic absorption spectroscopy (AAS) using a Shimadzu AA7000 (Shimadzu Corporation, Kyoto, Japan) spectrophotometer through flame ionization and continuous flow injection with an acetylene-air mixture gas source. The conditions regarding the detection of each element were adjusted automatically by the control software. The concentration of the elements was determined according to preliminary calibrations. (The LOD for Zn^2+^ and Pb^2+^ is 1 ppb).

In the case of selectivity studies, 23 different metal salts (with carbonate counterion: Rb^+^, Li^+^, Cs^+^; sulfate counterion: Mn^2+^, Fe^2+^; hydroxide counterion: Ba^2+^; chloride counterion: Sr^2+^, Al^3+^, Hg^2+^, Bi^3+^; iodide counterion: Cd^2+^; acetate counterion: K^+^, Ni^2+^, Co^2+^, Na^+^, Cu^2+^, Ag^+^, Ca^2+^, Zn^2+^, Mg^2+^, nitrate counterion: Cr^3+^, Pd^2+^, Pb^2+^) were added separately to the membrane-containing wells as 10^−5^ mol/L aqueous solutions.

Validation was carried out by measuring untreated water from the Danube River with the natural content of the studied metal ions. Detailed information regarding the composition of the river water can be found in the Supplementary Materials.

The plasma treatment was performed with an FG 5001 plasma generator (Plasmatreat GmbH, Steinhagen, Germany). The plasma was generated from compressed air (air pressure of 3.5 bar). The pressure was set by a gas reductor equipped with a manometer. The compressed air with the reduced pressure was introduced into a Plasmatreat RD1004 rotating plasma head. The parameters affecting the plasma properties (voltage of 280 V, and electric current of 17.5 A) were set on the digital control unit. The plasma head was mounted on a frame. The microtiter plate was treated by moving it under the plasma head at different linear speeds, which defined the time of the treatments. The vertical distance between the plasma head and the microplate was the second parameter.

Contact angle measurements were made with a smartphone camera, and software was used to determine the angles. A 10 µL water droplet was set in the chamber in front of the camera. The measurements were made at 25 °C and a humidity of 60%.

Attenuated total reflectance Fourier-transform infrared (ATR-FTIR) spectra were recorded on a PerkinElmer Spectrum Two FTIR (PerkinElmer, Inc., Shelton, CT, USA) with a universal ATR accessory head.

### 2.2. Determination of Limit of Detection and Response Time

Because of the inadequate linearity of the calibration curve, the detection limit (LOD) was determined based on a statistical data evaluation by using absorbance data of the UV/Vis calibration curve. For determining the signal-to-noise ratio, the absorbance of the membrane was measured in contact with distilled water, and the standard deviation of these blank measurements was determined from 12 independent measurements (n = 12, s = 0.009). Twelve separate measurements of the absorption were performed in low concentrations of the corresponding metal ion, and the averages of the measured absorbances were statistically compared with that of the blank by using a *t*-test. The LOD was defined as the lowest concentration that significantly differs from the blank signal of the membrane in distilled water at a significance level of 95% according to the following equation:

$$A_{LOD}=A_{distilled\;water}-t\left(95\%\right)\cdot \frac{s}{\sqrt{n}}$$

where *A_LOD_* is the absorbance signal, which belongs to the metal ion concentration of the LOD, *A_distilled water_* is the background absorbance in the case of measuring the distilled water sample, *t*(95%) is the Student parameter at a significance level of 95%, *s* is the sample standard deviation and *n* is the number of the samples. Therefore, if the measured absorbance is lower than or equal to the *A_LOD_*, it is significantly different from the background signal.

The response time was defined as the time required to reach 95% of a constant optical response. Response times were determined from 3 independent measurements. In the reported case, the optode membranes have limited permeability toward the sample solutions, and not all of the ionophores are active in chemosensing due to the former limitation and the adverse effects of covalent immobilization. When measuring ion concentrations above 10^−5^ mol/L, the reported response times refer to the non-equilibrium-type response induced by an interfacial phenomenon only involving the action of the membrane surface. Accordingly, additional ion-exchanging components were not copolymerized in the membrane; thus, permselectivity was also not provided.

## 3. Results and Discussion

### 3.1. Surface Modification of UV-Transparent Microplates

#### 3.1.1. Surface Activation

Initially, the chemically inert surface of the cycloolefin polymer of the UV-transparent microplate was activated by using atmospheric plasma treatment. According to a preliminary optimization, the treatment was carried out from a distance of 30 mm for 2 s. Moreover, a wet, chemical modification method was also tested in parallel using a so-called “Piranha-method” [43], which was previously described to polarize polyvinylidene difluoride (PVDF; detailed information about this method can be found in the Supplementary Materials). As a result of these surface modification techniques, the initially apolar (hydrophobic) and inactive polymer became polarized due to the introduced –OH and =O groups on the surface (both treatments induced various chemical and physical changes on the surface, but mainly the ones mentioned above are responsible for the polarization). Consequently, the relative amount of the easily modifiable OH groups can be estimated by contact angle measurements, which directly reflect the extent of polarization of the polymer surface. Thus, the surface-activation efficiency of the two applied methods was compared based on the decreases in contact angle values (Table 1).

As a result of the surface activation step, the initial 89° contact angle of water of the untreated polymer decreased by 7° using the Piranha method and by 29° applying the atmospheric plasma treatment. These values clearly indicate that the plasma treatment results in a more polarized polymer surface with higher wettability. This means that more functional groups can be grafted to the surface with the plasma treatment, making it a more efficient procedure for surface activation. Efficiency also depends on the time required for the treatment, which was 10 min for the Piranha method and only 2 s for the plasma activation.

#### 3.1.2. Silica Coating

**Figure 2:**
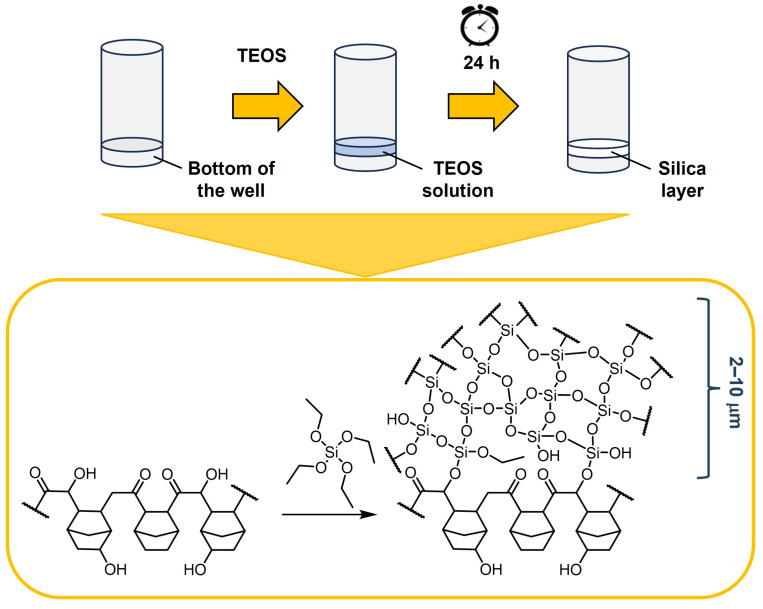
Formation of the silica coating layer on the surface of previously hydroxylated cycloolefin via the condensation of the TEOS monomer.

In the second step of the surface modification, the formation of a silica thin-layer was attempted using the commonly applied condensation method of tetraethyl orthosilicate (TEOS) monomers in aqueous media [44]. These experiments were carried out by incubating the pretreated well bottoms in a 200 µL solution of isopropyl alcohol containing 3 V/V% TEOS and 0.5 V/V% water. The reaction was incubated for 24 h at room temperature. As a result, 2–10 µm-thick silica layers formed. The optical properties of these plates were studied as a function of the layer thickness. Unfortunately, the silica coating strongly decreased the light transmittance of the plates (additional information can be found in the Supplementary Materials). This adverse effect was observed even in the case of the thinnest (2 µm) coating (Figure 2).

As the obtained absorbance values were close to the upper detectable limit, the component-specific response signal was suppressed during the optochemical detection. Accordingly, introducing a silica thin layer was not performed during further development, despite the fact that omitting the coating significantly reduced the achievable extent of labeling of the subsequently attached 3-(trimethoxysilyl)propyl methacrylate (TSPM) linker.

#### 3.1.3. Linker Docking

The functionalization process was continued via the direct immobilization of the TSPM linkers to the hydroxylated polymer surface. The pretreated wells were filled with the solution of the linker (100 µL/well; 3 V/V% TSPM, 1 V/V% pH = 3.0 HCl-water, 96 V/V% propan-2-ol) and then were incubated for 24 h at room temperature followed by washing with distilled water (3 × 100 µL/well). The extent of labeling was determined through the UV-calibration of different linker concentrations. (The solution of the unreacted linker was sampled, and its concentration was determined according to Lambert-Beer’s law. The difference from the initial concentration indirectly determines the amount of immobilized linkers per well. Further information can be found in the Supplementary Materials). The extent of labeling regarding the immobilized linker was 1.0 × 10^−6^ mol/well (±5.0%).

#### 3.1.4. Photocatalytic Copolymerization

The final step was the functionalization with the sensor molecules, which was carried out via photocatalytic copolymerization. The following monomer mixture was prepared in the dark: 50 mg acrylamide, 100 µL 2-hydroxyethyl methacrylate, 2.5 mg sensor molecule **1**, 50 µL 2-hydroxy-2-methyl-1-phenylpropan-1-one photoinitiator. The mixture was diluted 500-fold with acetone, and then, 100 µL of this stock solution was added to each well. The acetone was evaporated in the dark. The remaining thin film was subjected to UV irradiation [20 × 1 min with 1 min interruptions using a UV-light source (254 nm, 50 W) from a distance of 3 cm]. The composition of the polymer matrix was designed according to the reported analogous work [45,46,47,48,49], as similar polymer films have already been prepared successfully, while the polymerization process was carried out based on our previously reported procedure optimized for creating chemosensing optode films [49]. After the polymerization, the resulting membranes were washed sequentially with toluene (150 µL), methanol (150 µL) and distilled water (3 × 150 µL).

The average thickness of the sensing layer at the bottom of the wells was 10 µm (calculated based on the previously determined 1.1 g/cm^3^ density of the resulting copolymer and the amount of the monomer mixture). This layer contains 6.6 × 10^−9^ mol sensor molecule per well, which theoretically provides a binding capacity of 3.0 × 10^−4^ mol per well for the preferred ions (calculated for 1:1 complex stoichiometry and 20 µL of sample volume).

**Scheme 1:**
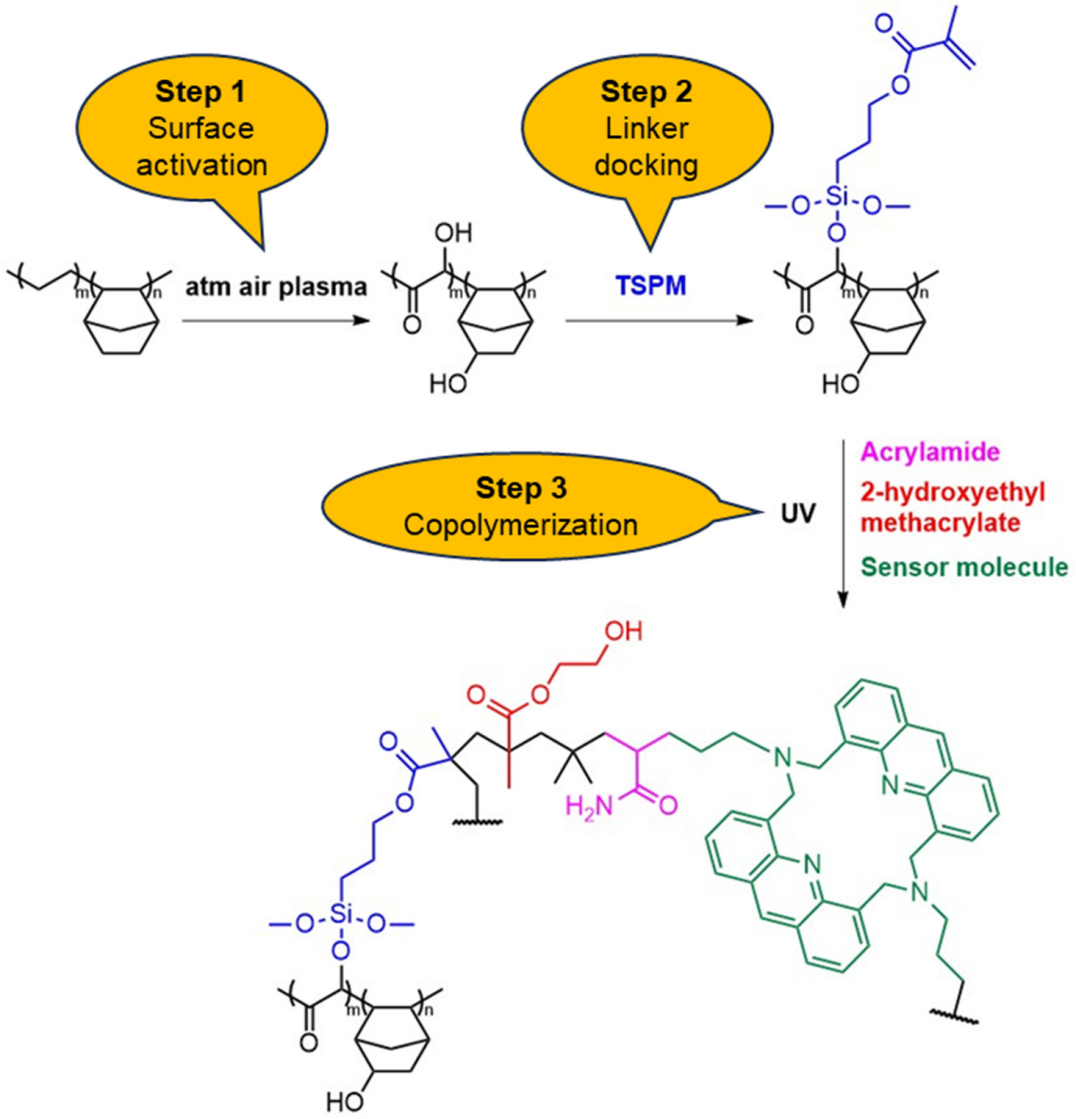
Procedure for functionalizing the polymer surface of the optosensing microplate device. (Only one of the possible chemical structures of the resulting polymer is shown. Constitution of the monomers, as well as the chemical and steric microenvironment of the sensor molecules, can vary diversely within the sensing membrane).

The synthetic procedure for surface functionalizing is shown in Scheme 1.

#### 3.1.5. Characterization of the Functionalized Microplate Surface

**Figure 3:**
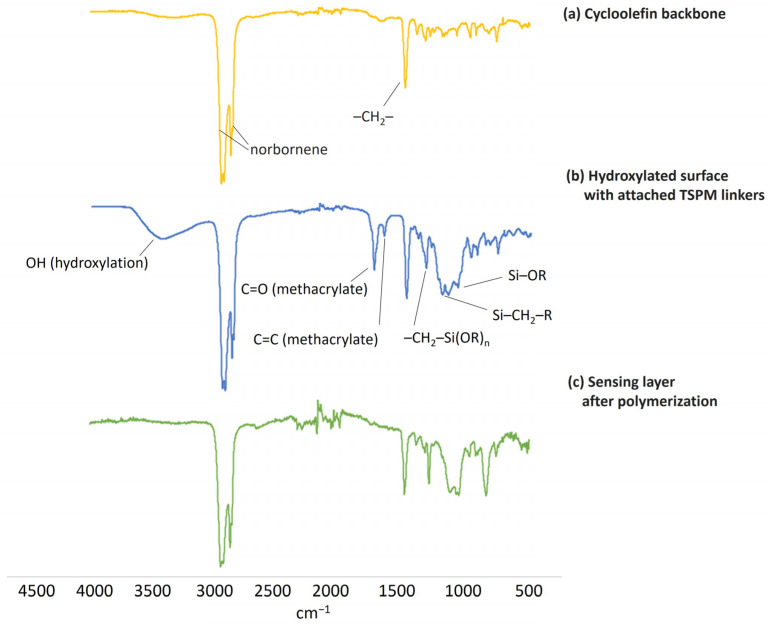
IR-spectra of the surface of the functionalized wells after each step of the synthetic procedure.

The functionalization process was monitored using the ATR-FTIR method (~100 µm signal-penetration depth). The results can be seen in Figure 3.

Part (a) of Figure 3 is the spectrum of the untreated cycloolefin and was consequential for the available reported results [50]. The absorption bands at 2866 and 2941 cm^−1^ are assigned to the sp^3^-C symmetric and asymmetric stretching vibrations of the norbornene units, respectively, while the intensive peak at 1455 cm^−1^ is attributed to –CH_2_– symmetric bending. The small-intensity peaks below 1000 cm^−1^ refer to the vibrations of the polymer backbone.

Part (b) refers to the polarized surface after introducing the TSPM linkers. The broad signal above 3000 cm^−1^ indicates the appearance of –OH groups. The other new peaks in the middle spectral region identify the linker. The characteristic peaks for the surface-attached alkoxysilane unit (1000–1110 cm^−1^ for Si–OR, 1265 cm^−1^ for Si–CH_2_–R and 1435 cm^−1^ for –CH_2_–Si(OR)_n_) and those of the methacrylate unit (1640 and 1720 cm^−1^ for C=C and C=O, respectively) are present [51,52].

After the polymerization, the spectrum of the treated surface was further altered, but it became quite complex to identify all the characteristic peaks. (Moreover, the ratio of the sensing layer and the polymer backbone was less than 1:5; thus, the signals of the backbone remained dominant.) The intensive peaks around 1200–1100 cm^−1^, corresponding to the stretching of the C–C–O and O–C–C units, overlap with the background signal [53]. Similarly, the characteristic –CH_3_ signals are also covered by those of the cycloolefin around 2900 cm^−1^, and the relative intensity of the methacrylate units decreased [53]. The broad signal of the OH group also disappeared, which is attributed to the observation that the polarization of the surface continuously decreased after the plasma treatment (similar observations were previously made [39]). The continuously increasing contact angle values [starting from the initial 89° of the untreated well (Table 1) up to 74°] indicated that the polarization effect of plasma treatment was reduced to a quarter after a 24 h storage of the plates in the air. This phenomenon is in accordance with the strongly decreased OH-signal in spectrum (c). The appearance of new small-intensity peaks on the fingerprint region (especially in the range of 700–800 cm^−1^ and around 500 cm^−1^) correspond to the aromatic stretching due to the presence of the sensor molecule. However, its presence is indicated more clearly by the UV spectrum of the modified wells.

**Figure 4:**
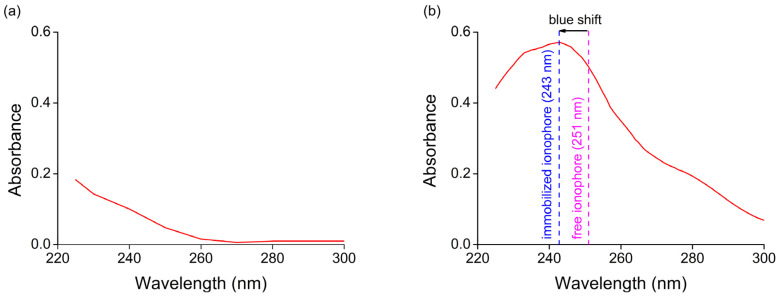
UV-absorbance spectrum of the pretreated (polarized, linker-functionalized) microplate well (**a**) and that of the sensing microplate containing the covalently immobilized ionophore **1** (**b**).

Before the immobilization of the sensor molecules, the activated wells showed only a slightly enhanced absorbance compared to the untreated ones (Figure 4).

After the immobilization of ionophore **1**, the appearance of its characteristic spectrum proved the presence of the host molecules inside the membranes. As the heterogeneity (limitations of pipetting, overheating during photocatalysis, etc.) and chemical diversity (options for the multifunctional monomers to form chemical bonds with a different constitutional order, steric diversity inside the bulk phase, heterogeneous microenvironment of the sensor molecules, etc.) of the obtained membranes do not allow for their exact characterization, Scheme 1 represents only one possible structural unit of the membrane. Furthermore, a certain proportion of the sensor molecules can be embedded into the polymer as inclusions of the monomer mixture without covalent immobilization. However, the slight hypsochromic shift of the characteristic absorption band of the sensor molecule 251 nm peak wavelength for the dissolved free sensor molecule [42], while that of 243 nm for the immobilized one; see part (b) in Figure 4 indicates covalent modification, and the lack of the removed ionophores upon careful extraction of the membranes also supports the assumption of the effective covalent immobilization.

**Figure 5:**
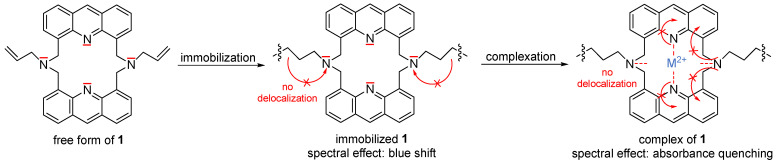
Structural and spectral changes upon immobilization and complexation of **1**.

Figure 5 represents the structural changes upon immobilization and complexation in a relationship with the caused spectral changes.

The optochemical response of **1** based on the internal charge transfer (ICT) mechanism is affected by every change in the microenvironment influencing the electronic structure, i.e., complexation, immobilization, etc. After excitation, the charge transfer is directed from the donor *N*-atoms of the receptor part toward the electron deficient chromophore unit as an acceptor.

In the case of covalent immobilization or upon complexation with a cation, the ability of the *N*-atoms of the receptor unit to donate electrons decreases significantly and the conjugation of their lone pairs with the *π*–system of the chromophore induces a change in the absorbance intensity, while the absorption spectrum shifts in the direction of shorter wavelengths.

Since the acridine *N*-atoms are parts of the chromophores, their altered electron density has the main effect on the ICT mechanism. However, as the aliphatic *N*-atoms of the receptor unit are at allyl-positions regarding the reactive terminal double bonds aiming to covalently immobilize the host molecule and at benzyl positions regarding the chromophore unit, the lone pairs of these *N*-atoms can be easily conjugated through the whole molecular structure. After immobilizing the host molecule via its allyl units, the extent of electron conjugation decreases and the energy requirement for the excitation of the entire electron system slightly increases, which is perceived as a blue shift in the absorbance spectrum.

In the case of the coordination of cations, the free lone pairs of all electron donor *N*-atoms participate in complexation; thus, their conjugation with the *π*–system ceases, and the absorbance decreases in accordance with the decreasing degree of delocalization.

### 3.2. Procedure for Measurement

**Figure 6:**
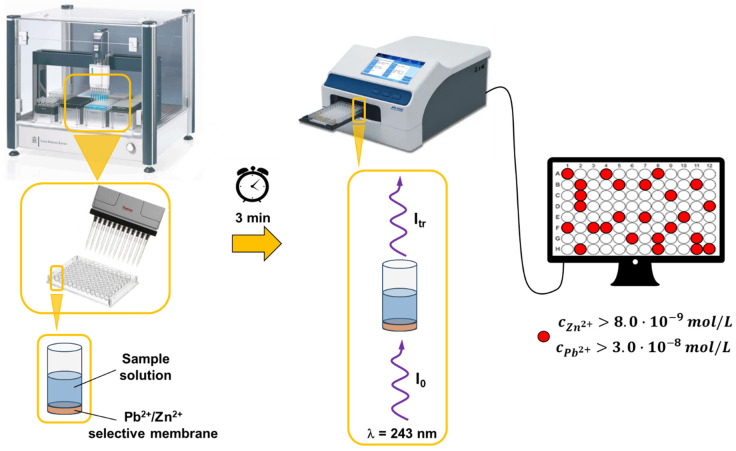
The applied measurement protocol for sample screening.

The Zn^2+^ and Pb^2+^ contents of samples can be measured by directly adding 20 µL (the lowest possible volume that can be evenly spread on the bottoms of the wells without any specific procedure) solutions to the microplate wells containing the sensing membrane on their flat bottoms. Even spreading can be supported by shaking with a plate bed, but it is not necessary for aqueous samples in general. If the sensing membranes are previously wetted (incubation with distilled water for 5 min), an incubation of 2 min was found to be enough before the absorbance measurements. During the incubation, a portion of the sensor molecules inside the upper surface layer of the sensing membranes forms complexes with the dissolved Zn^2+^ or Pb^2+^. As the complex formation results in a distortion of the electron system of the host molecule, it causes a concentration-proportional absorbance change in the polymer layer. (The inactive sensor molecules inside the polymer cause only a constant background signal.) The plate reader should be operated at a 243 nm peak wavelength instead of in scanning mode to accelerate the screening. After the absorbance detection, the samples can be removed through simple pipetting, followed by a washing procedure to remove the trace amounts of sample from the membrane surface and recover the original background signal of the plate. The measurement protocol is summarized in Figure 6.

It can clearly be seen from the applied protocol that it only enables the determination of the dissolved forms of the preferred ions since their diffusion, at least into the surface of the polymer, is essential. The ion concentrations shown in Figure 6 are the limit of detection values for the preferred ions, as the reported screening method is suitable for measuring samples with higher concentrations.

**Figure 7:**
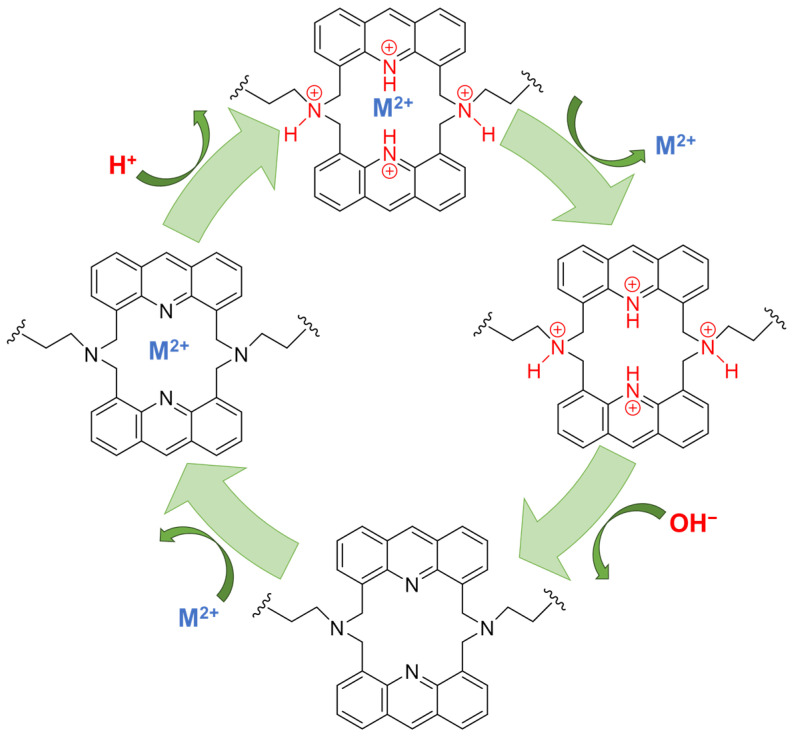
Acid-induced decomplexation of the immobilized sensor molecule (**1**) and its regeneration on the surface of the microplate wells.

After one screening, the microplate device can be used several times again. The regeneration, which means the removal of the complexed ions from the macrocycle cavity, can be performed by using a slightly acidic (aqueous solution of nitric acid with pH = 3.0) extraction; 150 µL of solution should be added, incubated for 2 min with bidirectional shaking on a plate-bed and removed by pipetting. Repeating the former procedure twice, followed by a third extraction with an aqueous sodium hydroxide solution with pH = 10.0 to convert ionophore **1** back to its neutral form, results in proper regeneration. The mechanism of the facile decomplexation of the sensor molecule can be seen in Figure 7.

A major advantage is that no chelating additives, e.g., EDTA, are needed for the decomplexation. At first, the slightly acidic medium protonates the basic tertiary-*N*-atoms and then the weakly basic acridine-*N* of the sensor molecule (**1**). This leads to the lost electron-donor ability of host molecule, which results in the dissociation of the sensor molecule–cation complex. Moreover, as only the sensor molecules inside the permeable surface layer are involved in the detection, regeneration is easier compared to plasticized bulk optodes.

### 3.3. Optical Response to Zn^2+^ and Pb^2+^

**Figure 8:**
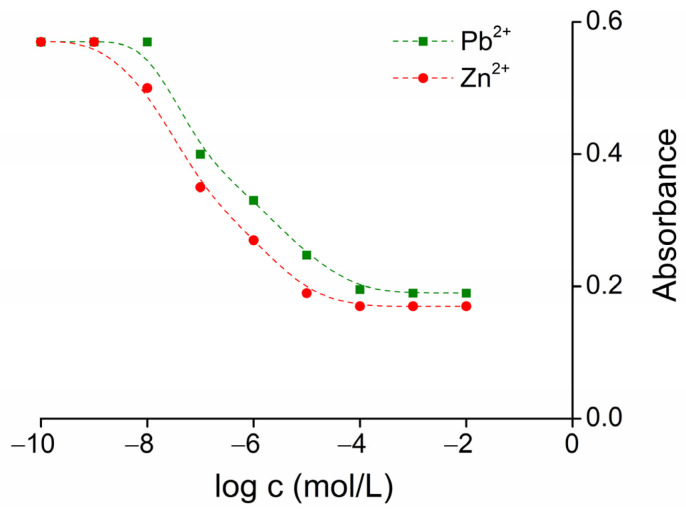
Calibration curves for UV/Vis titration of the functionalized microplate using single-component aqueous solutions of Zn^2+^ and Pb^2+^ at a 243 nm absorption-peak wavelength as a function of the logarithms of metal ion concentration.

Initially, titration was carried out by using single-component aqueous solutions of Zn^2+^ and Pb^2+^ in a wide concentration range. The obtained calibration curves can be seen in Figure 8.

The absorbance at the peak wavelength was reduced by about a third in the 10^−9^–10^−5^ mol/L concentration range. It is essential to point out that not all of the immobilized sensor molecules function as active binding sites. This is because of the numerous adverse effects on the ion-trap conformation inside the bulk polymer phase and the restricted membrane permeability towards the sample solution. The copolymeric structure of the sensing membrane carries many favorable properties in practice, e.g., easy regeneration, multiple usage, low chemical sensitivity, etc., but causes poor linearity of the dynamic range of the optical response. Therefore, the proposed method is mainly suitable for qualitative screening and not for quantitative determination of the detected metal ions, since only a rough estimation of their concentration can be made. The relative standard deviation was within 3.0%. The LOD was calculated as 8.0 × 10^−9^ mol/L for Zn^2+^ and 3.0 × 10^−8^ mol/L for Pb^2+^, which are significantly lower than the WHO acceptance limit of the corresponding metal ions (5 ppm [54] and 10 ppb [55] in drinking water, respectively); thus, the method is suitable for quality control even in the most strictly regulated drinking water samples.

**Figure 9:**
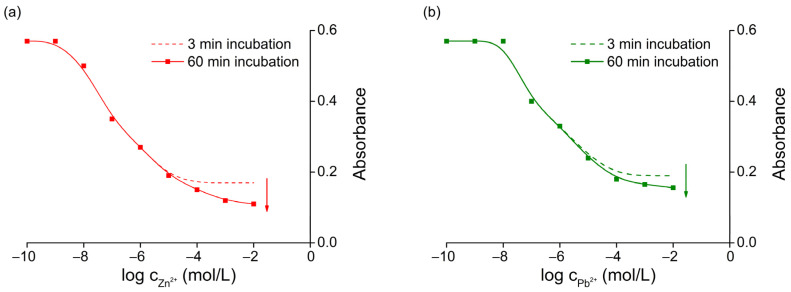
Time dependence of the optical response as a function of the metal ion concentration in single-component aqueous samples with Zn^2+^ (**a**) and Pb^2+^ (**b**) at 243 nm.

As the slope of the calibration curve decreased above 1.0 × 10^−5^ mol/L, quantitative estimations become impossible, but qualitative monitoring can still be performed. There are two reasons behind this altered slope of the calibration curve at higher Zn^2+^ and Pb^2+^ concentrations. One reason is the relative quantity of the sensor molecules inside the membrane (per well), which becomes equal to the full amount of the metal ions when the concentration of the samples reaches 3.0 × 10^−4^ mol/L (presuming that the full amount of the sensor molecules is involved in detection). Thus, the amount of immobilized ionophores needs to be increased to overcome the limited binding capacity of the membranes when optimizing for highly contaminated samples. The other reason is the limited permeability of the sensing membranes for the sample solutions, which would require a longer preincubation time before the measurements to establish the equilibrium concentration of the investigated metal ions between the membrane and the sample solution (diffusion control). Although the membranes are quite thin, the enhanced need for incubation is still necessary at high concentrations to reach complex-formation equilibrium, which is essential to provide a constant response signal. According to the general measurement procedure, the screening method only includes a 3 min preincubation of the samples on the membranes before initializing the absorbance screening. This was enough for samples with a metal ion concentration within the relevant LOD—10^−5^ mol/L range, but above 10^−5^ mol/L, the response time increased by orders of magnitude (Figure 9).

In the cases of high-concentration samples, 1 h of incubation was required to obtain a constant signal with an acceptable deviation. However, depending on the electrolyte content of the samples, even more time might be needed.

### 3.4. Study on Reversibility and Reusability

**Figure 10:**
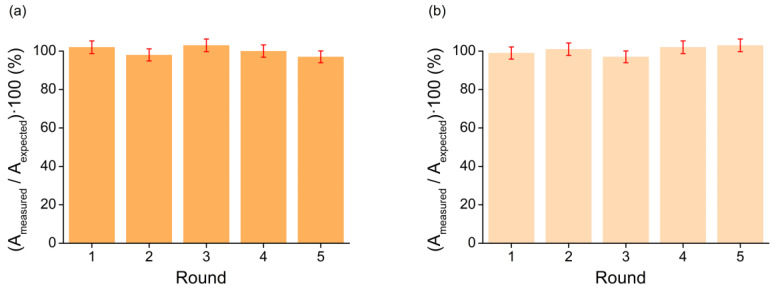
Reusability study of the membrane based on a measurement series of 1.0 × 10^−6^ mol/L aqueous Zn^2+^ samples (**a**) and real water samples (**b**) (*A_measured_*—absorbance value at 243 nm, *A_expected_*—calibration curve at the same wavelength).

In order to investigate the reusability of the reported sensing device, five parallel single-component aqueous samples with an identical concentration of Zn^2+^ (1.0 × 10^−6^ mol/L) were measured (part a of Figure 10). Moreover, to validate the reusability, repeated measurements were also carried out with real samples (part b of Figure 10; detailed information regarding the sample composition can be found in the Supplementary Materials). The regeneration procedure detailed in Section 3.2 was carried out between each measurement.

According to the results, only changes of less than 3.0% were measured in absorbance, because of the covalent immobilization and reversible complexation of the sensor molecules.

### 3.5. Study on Selectivity

**Figure 11:**
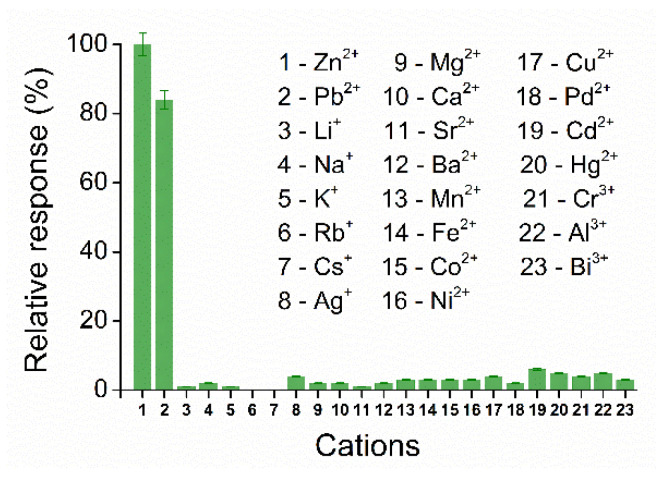
Separate selectivity studies of the membrane based on single-component aqueous solutions of metal ions (c = 1.0 × 10^−5^ mol/L) that may potentially compete.

There were also examinations in connection with selectivity in order to prove that the complexation behavior of the sensor molecule (**1**) is not affected by immobilization. The spectral change was detected in the equimolar presence of the above-mentioned metal salts (see Section 2.1) in single-component aqueous solutions (Figure 11).

It can clearly be seen that no significant change (within 5.0%) was caused, in the optical response, by the potential competing metal ions. Since Pb^2+^ is rarely expected to be co-present with Zn^2+^ in biological samples, this competition can only hinder the practical applicability in environmental monitoring. This might especially be the case for samples from mining areas or the close environment of landfills. This observation also indicates that the sensor molecules strongly coordinate the preferred ions even in the presence of Cd^2+^, Pb^2+^, Hg^2+^, Co^2+^, Ni^2+^ and Cu^2+^, which are considered typical competitors in similar chemosensing applications due to their soft cationic character.

### 3.6. Study on pH-Dependence

**Figure 12:**
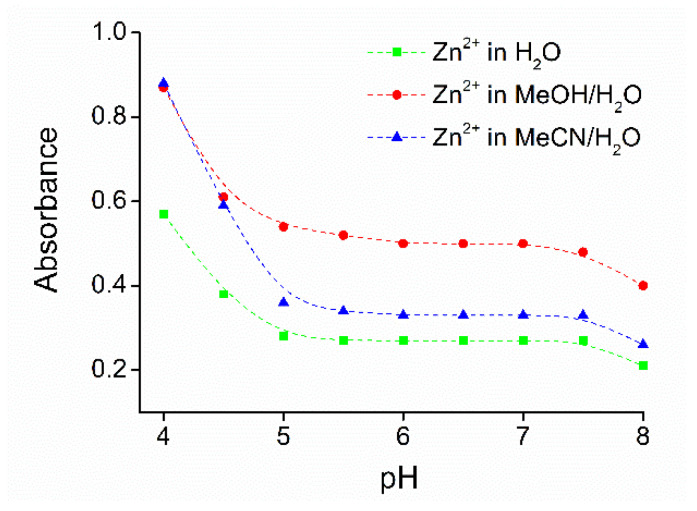
The pH-dependence of the optical response toward 1.0 × 10^−6^ mol/L Zn^2+^ at 243 nm in the case of aqueous samples; sample in methanol-water (9:1) and in acetonitrile-water (1:1) solvent mixtures (in organic solvents, the reported pH values refer to the initial pH of the aqueous part of the mixture).

The pH levels of the samples were changed by adding the appropriate amount of nitric acid or sodium hydroxide solutions into the wells containing aqueous samples of 1.0 × 10^−6^ mol/L Zn^2+^ or Pb^2+^. The results are depicted in Figure 12.

In the pH range from 5.0 to 7.5, constant absorbance was measured in aqueous samples. This range of pH values is the primary pH levels found in environmental and biological samples. As the pH drops below 5.5, the basic acridine-*N* of the sensor molecule (**1**) becomes protonated, which reduces the stability of the host–guest complex. This feature enables the regeneration process described in Section 3.2. The pH level above 7.5 is irrelevant as it leads to the precipitation of hydroxides of the favored cations.

The pH-dependence was strengthened in organic-solvent-containing samples due to the enhanced basicity of the nucleophilic amino groups within the macrocycle ring. The pH-independent working range decreased to the pH interval of 6.0–7.0 in these cases.

### 3.7. Detection in the Presence of Organic Medium

**Figure 13:**
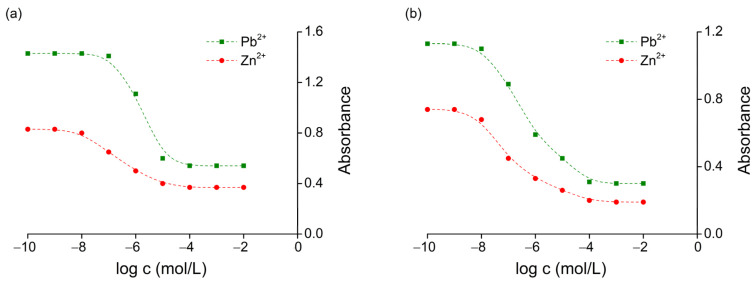
Calibration curves for Zn^2+^ and Pb^2+^ in single-component samples in (**a**) methanol:water (9:1) and (**b**) acetonitrile:water (1:1) solvent mixtures at a 243 nm absorption peak wavelength.

The covalent modification of the sensing microplate device also allows for its use for the analysis of dissolved ions in organic solvents. This would be impossible with conventional plasticized bulk membranes or physically immobilized ionophores. To investigate this advantageous property, screening was also carried out in organic-solvent-containing mixtures (Figure 13).

Compared to aqueous samples, the sensitivity (slope of the calibration curves) slightly decreased in general, while the LOD adversely increased to 9.0 × 10^−9^ and 2.0 × 10^−7^ mol/L in a methanol-based solvent for Zn^2+^ and Pb^2+^, respectively. In contrast, LOD values remained unchanged at 8.0 × 10^−9^ and 2.0 × 10^−8^ mol/L in an acetonitrile-based solvent for Zn^2+^ and Pb^2+^, respectively. The response time was also unchanged.

Overall, there are no significant limitations in applicability when replacing the aqueous media. Moreover, the method is quite indifferent to organic contaminants, a feature that is very important when extending the application for wastewater analysis.

### 3.8. Validation by Analyzing Real Samples

In order to verify practical applicability, untreated river water with natural Zn^2+^ and Pb^2+^ contents was measured. Detailed information regarding these samples can be found in the Supplementary Materials. Results were compared with those measured via ICP-OES (Table 2).

As can be seen, both the accuracy and precision are inferior to those of the generally applied instrumental analytical methods. Accordingly, the proposed probe is only suitable for giving a rough estimation of the dissolved form of the preferred metal ions, and the interference with Pb^2+^ is an obvious drawback when analyzing environmental samples. On the other hand, qualitative monitoring can be performed without any preparation, even for wastewater samples, if the solid particles were previously sedimented or filtered.

### 3.9. Analytical Performance and Throughput

The response time proved to be independent of the concentration of the preferred metal ions or the ionic strength of the sample above the LOD and below 10^−5^ mol/L. In the case of absorption scanning at the peak wavelength, measuring 96 samples on a microplate by recording five data points per sample takes 10 min, including the initial incubation, which means an overall throughput of screening of more than 1000 samples per hour (extrapolated from 96 measurements). It is important to mention that this performance was reached by using a basic-level plate reader. Naturally, throughput can be further improved by using more advanced instrumentation or by automating the whole process.

## 4. Comparison with Other Methods

The reported works on the selective optosensing of simple ions with a potential of reaching high-throughput were collected to put the obtained results into a context and support the evaluation of the properties of the proposed microplate-format sensing device (Table 3). In terms of instrument compatibility, the collected works are all suitable for measurements with a plate reader, which facilitates the speed of sample screening.

As it can be seen in the table, the reported sensor has the lowest limit of detection compared to other previously published microplate-format optodes, which enables the qualitative monitoring of Zn^2+^ and Pb^2+^ on a nanomolar scale. However, the dynamic working range is not as wide as in other cases making the optode less suitable for quantitative analysis. The response time of the membrane was 3 min, which is near average. A major advantage is that the reported membrane can be regenerated with a slightly acidic (pH = 3) aqueous solution and does not require any chelating additives. Moreover, compared to the other reported microplate-format optodes, it does not require sample preparation (e.g., buffering) when measuring real samples, such as river water.

## 5. Conclusions

**Figure 14:**
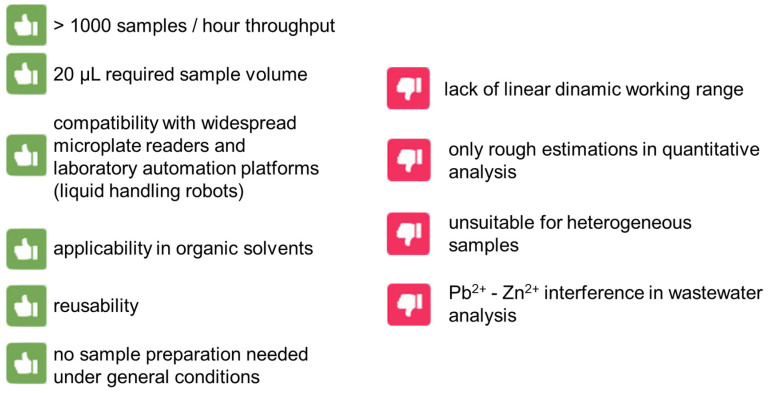
Highlighting the key features of the proposed method to specify its indication. (The performance per hour value was extrapolated from the time required for 96 measurements).

Our work provides an ultrafast sensing device with a high potential to replace conventional instrumental analytical methods in the high-throughput specific monitoring of dissolved Zn^2+^ and Pb^2+^. The designed covalently functionalized UV-transparent microtiter plate is compatible with any kind of generally applied reader spectrophotometers, as well as with conventional laboratory automation, e.g., liquid-handling workstations, plate transferring robots, multichannel pipetting devices. Thus, there is the possibility for its practical adaptation and full automation. The applicability of liquid samples for HTS was shown even for real samples, while satisfying the current requirements regarding the detection limits. In addition, we present a new procedure for the covalent modification of commercially available cycloolefin microtiter plates, which can also serve as a starting point for the future development of not only sensing devices, but various separation and assay tools for high-throughput experimentation. The main advantages and drawbacks of the proposed method are summarized in Figure 14.

The main drawback is that the proposed method is mainly suitable for qualitative screening and less suggested to apply for the quantitative determination of the detected metal ions, since only a rough estimation of their concentration can be made.

In summary, the reported studies revealed the competitiveness of the proposed chemosensing approach with other alternatives, while the presented plate-modification procedure can pave the way for future device-development with an emphasis on high-throughput experimentation.

## Figures and Tables

**Table 1:** Contact angles of the differently preactivated polymer surfaces at the bottoms of the microtiter plate wells.

	Contact Angle
Untreated well (reference)	89°
“Piranha-method”	82°
Atmospheric plasma treatment	60°

**Table 2:** Application of the optochemical sensing device for determining the Zn^2+^ and Pb^2+^ contents of untreated river water.

	Untreated River Water
$\mathrm{Real}\;c_{{Me}^{2+}}$ ^1^	50 ppb Zn^2+^ + 10 ppb Pb^2+^
$A{\;}_{theoretical}$ ^2^	0.22
${\bar{A}}_{observed}$	0.24
$\mathrm{Determined}\;c_{{Me}^{2+}}$ ^3^	260 ± 63 ppb

^1^ The real concentration of Zn^2+^ and Pb^2+^ measured via ICP-OES. ^2^ The theoretically expected cumulative absorbance for Zn^2+^ and Pb^2+^ at 243 nm based on its concentration measured via ICP-OES. ^3^ The concentration of Zn^2+^ was calculated according to the reported calibration curve (Section 3.3).

**Table 3:** Main operating characteristics of the reported plate-format optochemical probes for simple ions.

Target Ion	Detection Mode	Dynamic Working Range (mol/L)	LOD (mol/L)	Response Time (min)	Regenerability	Real Sample Type	Sample Preparation	Reference
Na^+^	A ^1^	NR ^3^	NR ^3^	5	NR ^3^	Blood serum	Buffering	[25]
K^+^		NR ^3^	NR ^3^	5	NR ^3^			
F^−^	A ^1^	$1.0\cdot 10^{-7}–1.1\cdot 10^{-3}$	$1.0\cdot 10^{-7}$	1–10	Single-use	Mouth rinse	Buffering	[30]
Cu^2+^	F ^2^	$1.0\cdot 10^{-7}–1.0\cdot 10^{-4}$	$1.0\cdot 10^{-8}$	3–100	With EDTA	Tap water	Buffering	[31]
Zn^2+^	F ^2^	$2.5\cdot 10^{-4}–1.8\cdot 10^{-3}$	$1.7\cdot 10^{-4}$	NR ^1^	NR ^3^	NR ^3^	Buffering	[32]
Pb^2+^	F ^2^	$4.0\cdot 10^{-7}–1.0\cdot 10^{-2}$	$4.0\cdot 10^{-7}$	<0.17	With distilled water	NR ^3^	No need	[33]
Zn^2+^	A ^1^	$8.0\cdot 10^{-9}–1.0\cdot 10^{-5}$	$8.0\cdot 10^{-9}$	3	With acidic solution	River water	No need	present work
Pb^2+^		$3.0\cdot 10^{-8}–1.0\cdot 10^{-5}$	$3.0\cdot 10^{-8}$	3				

^1^ A means absorbance. ^2^ F means fluorescence. ^3^ NR means not reported.

## Data Availability

The authors confirm that the data supporting the findings of this study are available within the article and its Supplementary Materials.

## Supplementary Materials

The following supporting information can be downloaded at: https://www.mdpi.com/article/10.3390/s24144529/s1, S1. Supplementary material regarding ICP-OES measurements; S2. Composition of river water samples; S3. “Piranha-method” for activating the surface of the cycloolefin wells; S4. UV calibration for determining the concentration of the remaining TSPM in the functionalizing solution; S5. Increased absorbance of the microplate wells containing a silica coating layer.
