# Supplementary material for: Covalently Modified Molecular-Recognition-Capable UV-Transparent Microplate for Ultra-High-Throughput Screening of Dissolved Zn2+ and Pb2+
Source: Sensors (Basel). 2024 Jul 12;24(14):4529. doi: 10.3390/s24144529 (PMC11281066; doi:10.3390/s24144529)
Supplement: Supplementary file 1 [file sensors-24-04529-s001.zip › sensors-3073360-supplementary.pdf]

Supplementary Materials

Covalently modified molecular recognition-capable UV-transparent microplate for ultra-high-throughput screening of dissolved Zn^{2+} and Pb^{2+}

Bálint Árpád Ádám [1], Bálint Kis-Tót [2], Bálint Jávor [1], Szabolcs László [3,4], Panna Vezse [1], Péter Huszthy [1], Tünde Tóth [1,5,*] and Ádám Golcs [1,2,6,*]

[1] Department of Organic Chemistry and Technology, Budapest University of Technology and Economics, Szent Gellért tér. 4., H-1111 Budapest, Hungary
[2] Department of Pharmaceutical Chemistry, Semmelweis University, Hőgyes Endre utca 9., H-1092 Budapest, Hungary
[3] Department of Inorganic and Analytical Chemistry, Faculty of Chemical Technology and Biotechnology, Budapest University of Technology and Economics, Műegyetem rkp. 3., H-1111 Budapest, Hungary
[4] Computation-Driven Chemistry Research Group, HUN-REN, Műegyetem rkp. 3., H-1111 Budapest, Hungary
[5] HUN-REN Centre for Energy Research, Konkoly-Thege Miklós utca 29-33., H-1121 Budapest, Hungary
[6] Center for Pharmacology and Drug Research & Development, Department of Pharmaceutical Chemistry, Semmelweis University, Hőgyes Endre utca 9., H-1092 Budapest, Hungary
* Correspondence: toth.tunde@vbk.bme.hu (Tünde Tóth), golcs.adam@semmelweis.hu (Ádám Golcs)

Contents

S1. Supplementary material regarding ICP-OES measurements

S2. Composition of river water samples

S3. 'Piranha-method' for activating the surface of the cycloolefine wells

S4. UV-calibration for determining the concentration of the remaining TSPM in the functionalizing solution

S5. Increased absorbance of the microplate wells containing a silica coating layer

S1. Supplementary material regarding ICP-OES measurements

Sample solutions were measured in simultaneous, multielement mode by a Labtest Plasmalab ICP-spectrometer (Labtest Equipment Company, USA) with a 40-channel Paschen-Runge vacuum polychromator with photomultiplier detectors, using 27 MHz argon plasma. Instrument settings were the following, forward power: 1.3 kW, sample introduction with a “OneNeb” nebulizer (Agilent Technologies, USA), cyclonic spay chamber and Gilson peristaltic pump (Gilson Company, USA) at 1 mL min^{-1} sample flow rate, observation height: 13.5 mm, integration time: 5 s. Limit of quantitation for Ag was 0.025 $mg{\cdot}L^{-1}$, (wavelength: 328.068 nm), for Ca was 0.03 $mg{\cdot}L^{-1}$, (wavelength: 422.673 nm), for Co was 0.019 $mg{\cdot}L^{-1}$, (wavelength: 238.892 nm), for Cu was 0.025 $mg{\cdot}L^{-1}$, (wavelength: 224.700 nm), for K was 0.05 $mg{\cdot}L^{-1}$, (wavelength: 766.491 nm), for Mg was 0.0005 $mg{\cdot}L^{-1}$, (wavelength: 279.553 nm), for Na was 0.19 $mg{\cdot}L^{-1}$, (wavelength: 589.592 nm), for Pb was 0.13 $mg{\cdot}L^{-1}$, (wavelength: 220.353 nm), and for Zn was 0.005 $mg{\cdot}L^{-1}$, (wavelength: 213.856 nm).

S2. Composition of river water samples

The river water samples were collected from river Danube in Budapest (GPS coordinates: 47°29'06.5"N 19°03'11.8"E, 2021.02.16. $10^{\underline{00}}$). The metal ion compositions were determined by ICP-OES method after filtering and acidifying the samples by nitric acid (**Table S1**).

Table S1. Composition of the investigated river water samples determined by ICP-OES

Elements	River water (mg/L)
Ag	<0.02
Al	<0.09
As	<0.07
B	<0.01
Ba	0.03
Be	<0.01
Bi	<0.19
Ca	59.8
Cd	0.01
Co	<0.02
Cr	<0.02
Cu	<0.02
Fe	0.07
Hg	<0.01
K	3.21
Li	<0.06
Mg	12.20
Mn	<0.01

Mo	<0.02
Na	19.9
Ni	<0.04
P	0.43
Pb[1]	0.01
S	12.5
Sb	<0.13
Se	<0.23
Sn	<0.08
Sr	0.23
Ti	<0.01
V	<0.02
W	<0.09
Zn[1]	0.05
Zr	<0.02

[1] The reported sensing device is capable of detecting Pb^{2+} and Zn^{2+}.

S3. 'Piranha-method' for activating the surface of the cycloolefine wells

To each well of the microtiter plate 20 μL of cc. H_2O_2 and 20 μL of cc. H_2SO_4 were added. The reaction was incubated for 10 min at room temperature, then the wells were washed twice with 100 μL distilled water. The effect of the reaction was studied by measuring the optical transparency of the wells. The treatment did not significantly influence the absorbance of the plate.

S4. UV-calibration for determining the concentration of the remaining TSPM in the functionalizing solution

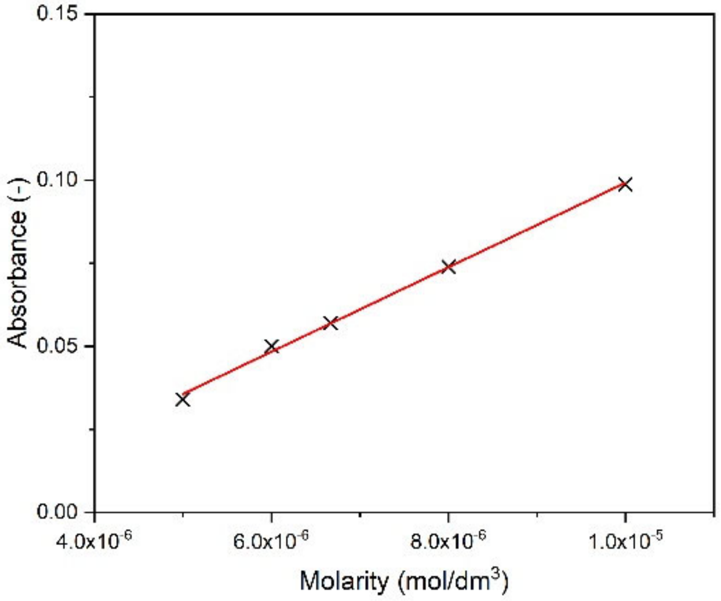

Figure S1. UV calibration curve for TSPM from from 5×10^{-6} to 10^{-5} mol/L

S5. Increased absorbance of the microplate wells containing a silica coating layer

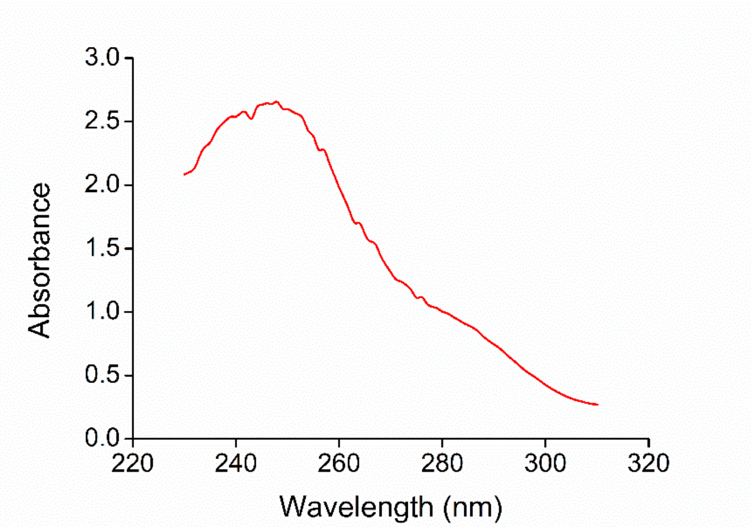

Figure S2. Absorbance by measuring 20 μL of distilled water on microplate wells containing 2 μm thick coating silica coating layer

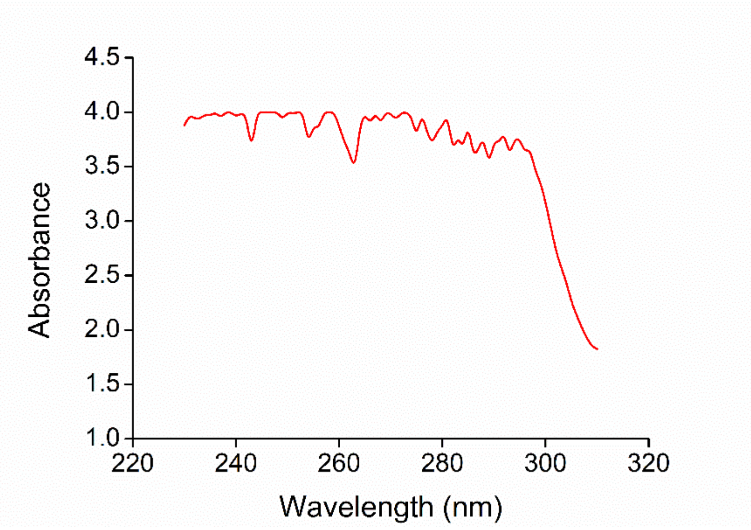

Figure S3. Absorbance by measuring 20 μL of distilled water on microplate wells containing 10 μm thick coating silica coating layer
